# The Speed of Sound in Silk: Linking Material Performance to Biological Function

**DOI:** 10.1002/adma.201401027

**Published:** 2014-06-06

**Authors:** Beth Mortimer, Shira D Gordon, Chris Holland, Clive R Siviour, Fritz Vollrath, James F C Windmill

**Affiliations:** University of Oxford Department of ZoologyOxford, OX1 3PS, UK; University of Strathclyde Department of Electronic and Electrical EngineeringGlasgow, G1 1XW, UK; University of Sheffield Department of Materials Science and EngineeringSheffield, S1 3JD, UK E-mail: christopher.holland@sheffi eld.ac.uk; University of Oxford Department of Engineering ScienceOxford, OX1 3PJ, UK

**Keywords:** signalling, resonance, vibration, web, silk

Whilst renowned for exceptional mechanical properties,[1] little is known about the sonic properties of silk. This is surprising given its widespread use by the spider for remote sensing and communication, as well as current industrial research efforts in the production of multifunctional materials.[2,3] To address this gap in our knowledge and provide further bioinspiration, this paper presents a systematic study confirming the physical basis of spider silk's sonic properties through a unique combination of laser vibrometry and high-rate ballistic impact. We report that modification of silk's modulus allows the spider to finely control the sonic properties: achieved either actively by spider spinning behavior or passively in response to the environment. Interpreting our results in the context of whole webs, we propose silk fibers are “tuned” to a resonant frequency that can be accessed through spider “plucking” behavior, which enables them to locate both prey and structural damage. Through comparison to cocoon silk and other industrial fibers, we find that spider dragline silk has the largest wavespeed range of any known material, making it an ideal model for fabrication of adjustable, green multifunctional materials.

The focus of this paper is dragline, or major ampullate silk, which exhibits exceptional mechanical performance as web scaffolding, but also forms the primary communication material of the orb web by signalling vibrations.[4–6] As these are multipurpose fibers, we suspect there may be as yet undefined evolutionary trade-offs that may limit the optimisation of mechanical and sonic properties – important information to consider when using spider silks as inspiration for our own materials.

Hence, understanding the physical basis of spider silk's sonic properties will be key to understanding the evolutionary interactions between mechanical and signalling performance. Fibers propagate both longitudinal (compression/tension) and transverse waves,[7] where the former consists of vibrations along the fiber length, and the latter those perpendicular to the fiber.[8] Theory shows that the longitudinal wavespeed is determined by material properties, whereas transverse wavespeed is additionally governed by applied tension.[9] However, these mechanical properties are complex, and to-date, experimental measurements and analyses have not fully elucidated wave propagation behavior in silks.

The sonic properties of a spider's silk and web are difficult to measure in Nature as the vibrational ‘landscape' is highly complex involving the geometry of interacting silk strands of different tensions and types.[10–12] Previous studies have measured wavespeeds in parts of the web using Brillouin light scattering, and web propagation speeds using laser vibrometry, both showing variable results.[5,13] Vibration propagation distance has also been measured in webs, showing lower attenuation of longitudinal compared to transverse waves.[5,14,15]

Given the apparent complications in experimental measurements of wave propagation in webs, we here investigate silk fibers independent of the web, allowing accurate matching between material and vibrational properties. Our study combines physical theory with the complementary experimental techniques of laser vibrometry and ballistic impact to confirm the physical basis of the sonic properties of a range of materials. By comparing spider silk to other materials, we can infer the constraints on the evolution of signalling properties in terms of material structure. Where these limitations are apparent, we discuss the means that the spider might employ to adjust the balance between structural support and signalling functions. The experimental techniques presented here provide novel contributions towards understanding complex web vibration and spider evolution and our approach provides important insights into Nature's design of stimuli-responsive multifunctional polymeric materials.

In order to confirm the applicability of basic wave equations to the physical basis for sonic properties of spider silk fibers, longitudinal and transverse wavespeeds are first experimentally measured using laser vibrometry and high-rate ballistic impact. These results are then placed within the context of a range of materials tested using laser vibrometry (alongside our previous high rate ballistic impact work [16] before they are all compared to their theoretical values (**Figure** **1**).

**Figure 1:**
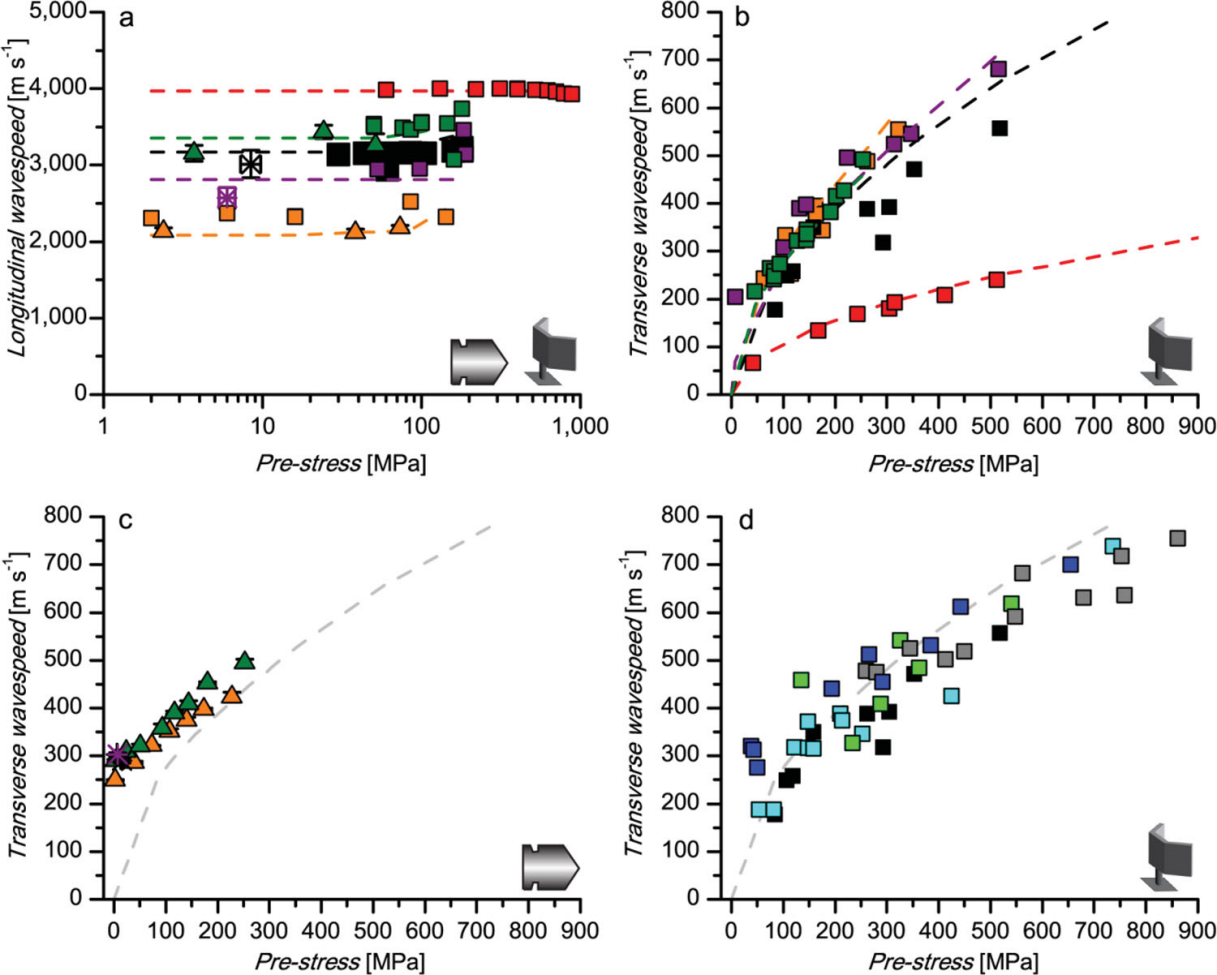
Wavespeed as a function of pre-stress. a) Longitudinal wavespeed (log axis) and b)-d) transverse wavespeed. Theoretical calculations are given by dashed lines, vibrometry data (shown by speaker symbol) by squares, ballistic impact data (shown by bullet symbol) for spider silks at low tensions and 220 m s^–1^ by stars and data adapted from Drodge et al.[16] by triangles. Ballistic impact data include standard error of the mean bars, for both wavespeed and pre-stress for spider silks, and wavespeed for nylon and silkworm silk as pre-stress was consistent between repeats. Acoustic measurements do not have error bars as they are all individual samples. Materials: copper beryllium wire (red), silkworm silk (dark green), nylon (orange), *Nephila* major ampullate (MA) spider silk (big size, black; small size, dark grey; small size supercontracted, blue), *Nephila* minor ampullate (MiA) spider silk (purple), mixture of *Nephila* silks (2 MA, 2 MiA: cyan), and *Araneus* spider bundle (2 MA, 2 MiA: green). For b) and c), the grey dashed line gives a reference spider silk theoretical curve.

The longitudinal wavespeed for all materials tested was calculated from the two independent experimental techniques and shows close agreement to theory, thus validating our approach (Figure 1a). The ballistic impact high-rate data show some small differences when compared to the vibrometry data and theory, explained by artefacts in the way in which longitudinal wavespeed is calculated: it is dependent on the gradient of the stress-strain curve, so small errors in the ballistic impact high-rate stress-strain coordinates are converted to larger errors in the modulus and wavespeed.[16] Additionally, error may be introduced from fluctuations in cross-sectional area.[17] A comparison of the quasi-static and ballistic impact high-rate stress-strain co-ordinates modified from Drodge et al. (2012) is given in the Supporting Figure S1.[16]

The longitudinal wavespeed (*C_L_*) in a fiber is given by:


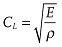
(1)

Where *E* is the storage modulus, a measure of the spring or purely elastic stiffness, and *ρ* is the density. As silks and nylon have similar densities due to comparable polymeric structures, storage modulus is key to the differences in longitudinal wavespeed. In terms of the consistency of signalling, indicated by a fiber's response to tension, longitudinal wavespeed in both natural silks and synthetic polymers increases in response to increasing static pre-stress above ∼50 MPa, by up to 16 % by 200 MPa (range 6 to 16 % for the different materials). This is because the storage modulus has a shallow linear relationship with pre-stress.[18,19]

The transverse wavespeed (*C_t_*) is given in theory by:


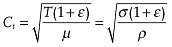
(2)

Where *T* is the tension on the fiber, *ε* is the strain on the fiber, *μ* is the mass per unit length of the unstretched fiber and *σ* is the fiber stress. The dependence of transverse wavespeed on stress is therefore similar for all polymeric materials tested (Figure 1b-d), due to their similar densities. Transverse waves are less consistent than longitudinal waves as their wavespeed is sensitive to pre-stress, showing an increase in wavespeed of up to 102% from 50 to 200 MPa (range 83 to 102 % for the different materials).

Although the transverse experimental data support the application of the theoretical equations in this context, there are some deviations. For example, the theory applies to standing waves specifically, and therefore describes the acoustic data well (Figure 1b and d). The ballistic impact high-rate data do not intercept at zero as the transverse waves are propagating rather than standing (Figure 1c). Even at zero pre-tension, there is a longitudinal pre-cursor wave that propagates ahead of the transverse wave and causes additional stress in the fiber.[16]

In summary, the measured longitudinal and transverse wavespeeds for major and minor ampullate silk are 2940–3230 and 117–557 m s^-1^ respectively, comparable to previous theoretical calculations on *Nephila* silks (longitudinal 2111–2183 m s^-1^, transverse 109–421 m s^-1^).[5,9] However, previously measured vibration propagation speeds in *Nephila* webs are slower (longitudinal 107–1070 m s^-1^, transverse 70-100 m s^-1^; frequencies of forced waves 10-1000 Hz).[5] We propose this to be due in part to material dispersion of the relatively low-frequency, high-amplitude forced vibrations used in the previous study on webs (see Supporting Discussion and Supporting Figure S2), and also practical difficulties in measuring web silk fibers and keeping factors such as tension constant during the contact vibration used in these studies.

To better understand the evolution of spider silk as a signalling material, we have quantified the sonic properties of single fibers of silks independent of the web, thus permitting the physical basis of sonic properties to be confirmed for the first time. The consistency of signal transmission and information transfer is further explored in the Supporting Discussion and Supporting Figures S3 and S4. Combined, these data provide interesting insights into the evolution of spider silks' sonic properties.

The transverse wavespeed of spider silk is determined primarily by its density, and hence differs little from other polymers. Density depends on the material structure, which constrains the shaping of transverse wavespeed by natural selection. In contrast, the value of the longitudinal wavespeed is tuned by the storage modulus, or spring stiffness, which differs between the materials tested. Therefore, in principle, the storage modulus could be selected in spiders to adapt the longitudinal sonic properties. However, storage modulus also dictates mechanical properties, so the wavespeed will be constrained by the dual functions of the storage modulus (see below).

Differences in signalling consistency of transverse and longitudinal waves allow us to infer their possible roles in the web (**Table** **1**). Longitudinal waves have greater scope for signalling uses as they propagate reliable, damage tolerant signals to the spider regardless of the tension, humidity and strain history of the web. This supports previous studies of webs showing lower attenuation for longitudinal compared to transverse waves, explained by lower external damping and minimal wave reflection at junctions.[5,9,14,15,20] As attenuation has direct effects on wave amplitude, longitudinal waves may therefore aid spider vibration detection in the web and possibly discrimination of signals.[5,6,15] This difference in consistency and attenuation may explain behavioral data showing that spiders are more likely to respond to web-borne longitudinal waves.[6,21,22]

**Table 1:** Summary of findings

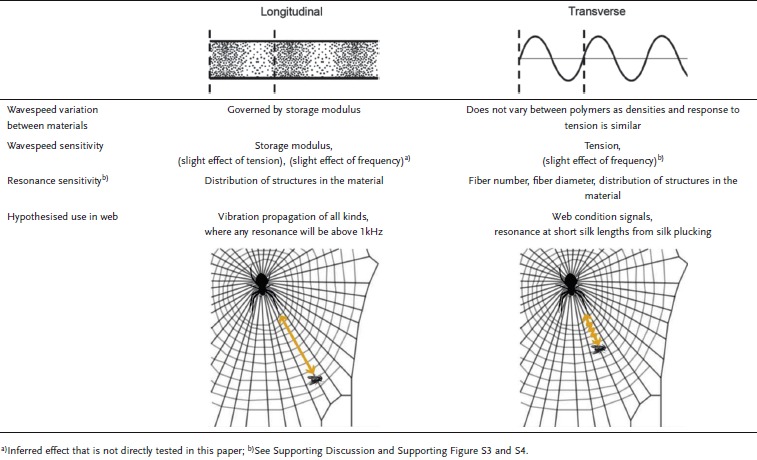

In contrast, transverse waves are highly sensitive to small changes in tension, meaning that the transverse wavespeed is difficult to control, and is affected by both passive mechanisms, including wind, caught objects and even humidity conditions, but also active mechanisms such as web pre-tension and number of fibers. The sonic properties may be a disadvantage in terms of consistent signalling, but could be an advantage for real-time probing of web condition (e.g. damage and tension) via reflection due to changes in impedance.[5,15]

Interpreting our measured wavespeeds within the context of *Nephila's* web geometry, possible frequency ranges for the different wave types in a web can be calculated. For a 0.7 m web under tension,[11,23] the minimum resonant frequencies are 1150 Hz for longitudinal and 75 Hz for transverse waves, assuming the vibrating source is the maximum displacement point of the wave.

This calculation further supports the use of longitudinal waves over transverse waves for information gathering in webs as longitudinal waves will not resonate close to prey-generated vibration frequencies (typically under 1 kHz).[5,12,22] This means that silk resonance is unlikely to interfere with prey-driven vibrations. This may be an advantage for detecting diverse and inconsistent vibration signals, particularly the broadband signals produced by prey.[5,21] Therefore, longitudinal resonance at high frequencies may have a selective advantage over resonance under 1 kHz.[5] This would enable prey-generated signals to propagate without resonant amplification, which may incur additional processing by the spider prior to interpretation.

Together, these interpretations may provide an explanation as to why spider vibration sensors have the highest displacement sensitivity at 1 kHz (∼100 nm)[7,24] in spite of prey not causing vibrations at such high frequencies.[5,12,22] The answer stems from the curious behavior of silk plucking by spiders; our results predict that following silk-plucking, longitudinal (and potentially transverse) waves will resonate close to or above 1 kHz at web-like lengths.[5,6,25] In principle this could be used to determine an objects' (or ‘intruders'’) radial thread location, as the frequencies will be higher on threads with a reflection point.

Moving beyond the web and comparing silk to other materials, we find that spider silk is a superb example of a high fidelity, tunable, environmentally responsive, multifunctional fiber. To facilitate comparison, we present a performance map of silks akin to Ashby plots, comparing the consistent and controllable longitudinal wavespeed (**Figure** **2**).[26]

**Figure 2:**
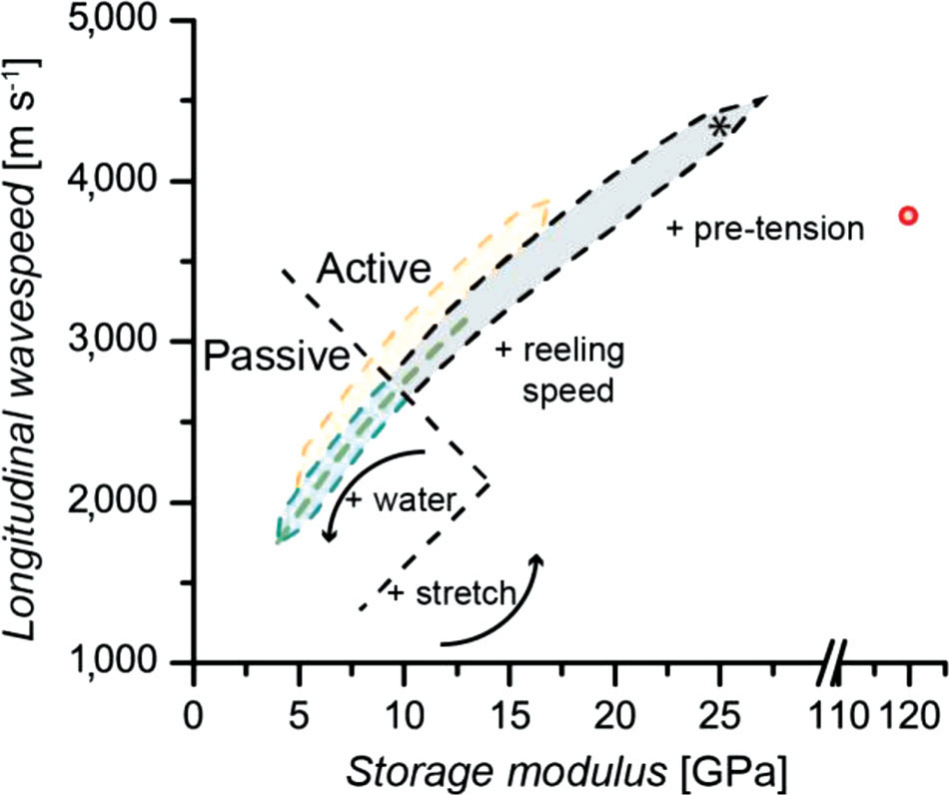
Storage modulus versus longitudinal wavespeed for different materials. Polymers and silks follow a (root) dashed line,[18] so shaded area is given to allow different materials to be distinguished. Metal wire has a single coordinate. Materials: copper beryllium wire (red), silkworm silk (dark green dashed line), nylon (orange), *Nephila* major ampullate spider silk (dry: black, wet: cyan). Asterisk gives low tension, dry Aciniform spider silk.[33] For the major ampullate silks, wavespeed can change with passive and active controls: for the former, wetting the silk allows supercontraction, lowering modulus; in the latter, modulus can be altered by processing conditions such as reeling speed, but also by applying tension or stretching, which increases the storage modulus to the highest levels measured.

Spider major ampullate silks occupy a unique niche of properties. Whereas a metallic alloy has one modulus and so one longitudinal wavespeed, polymers have a range, shaped by the sensitivity of their storage moduli to tension.[18,19] Of particular significance is our discovery that spider major ampullate silk has the largest longitudinal wavespeed range out of any known material due to the range of moduli available (3 to 30 GPa).[18,27,28] At the high end, similar to other polymers and silks, the storage modulus can be increased by tension and other processing conditions, such as the speed of silk production (i.e. spinning rate).[18,27,29] This provides active controls for the spider to alter the storage modulus and with that, the resulting wavespeed and mechanical properties of its silks. At the lower end, wetting major ampullate silk causes supercontraction, drastically decreasing modulus.[27,30] This provides a passive control, which can reset properties periodically, for example when dew descends at night.[31] Importantly, following the passive supercontraction, the silks can be actively stretched to again increase their moduli.[32] Spiders can therefore choose the extent to which the silks are stretched, giving them access to the whole range of moduli through their behavior, allowing them to shape the balance between mechanical and signalling properties as required.

In conclusion, by studying vibrations in spider silks in comparison with other materials, we can identify evolutionary trade-offs between mechanical and signalling functions, both governed by storage modulus. Storage modulus, in turn, may be adjusted actively by the spider via its spinning behavior in response to abiotic and biotic conditions, or it may be passively affected by climatic factors, such as humidity. Our approach to this phenomenon is interdisciplinary and by combining experimental, empirical and theoretical investigations, is not only beginning to elucidate vibration transmission in multifunctional materials, but will also be applicable far beyond our silk model system to a wider range of multipurpose biomaterials that aim to balance both signalling and mechanical functions. In this way we hope that spider silks may provide interesting and important insights beyond the specific adaptations of the web to provide ever more inspiration in the design of stimuli-responsive smart materials.[3]

## Experimental Section

Spider silks were obtained through forced reeling of spiders.[34] Two orb weaver species, *Nephila edulis* and *Araneus diadematus* were reeled for their silks under recorded lab conditions (∼21 °C, 40% relative humidity) at a speed of 20 mm s^–1^ onto cardboard frames of varying sizes (see Supporting Methods). Being invertebrates all spiders were handled according to local lab risk assessments/institutional ethical guidelines and do not currently fall under regulation by the UK Home Office or EU legislation. Other materials were also mounted loose onto cardboard frames: mulberry silkworm *Bombyx mori* cocoon silk, medium tenacity nylon and copper beryllium wire.

To control tension during vibrometry experiments, specimens were clamped into a Deben Microtest tensile stage (2 N; Supporting Figure S5). Silk fibers were vibrated using sound, amplified (TA-FE370, Sony) and transmitted from a loudspeaker (ESS Air Motion Transformer) that was positioned either perpendicular or 30 ° relative to the silk fiber axis for transverse and longitudinal waves respectively (Supporting Figure S6). A reference microphone (Bruel & Kjaer 4138) was positioned near the silk, perpendicular to the speaker. A ∼55 dB sound pressure level broadband linear chirp of frequencies 1–30 kHz was generated by the micro-scanning Laser Doppler Vibrometer system (PSV 300, Polytec), which measured the silk's nanometer movement with a fitted close-up unit (OFV 056). The noise floor of the vibrometer system was typically 0.3 μm s^-1^ (48 pm) for 1 kHz increasing to 3 μm s^-1^ (16 pm) for 30 kHz. The maximum vibration amplitude measurement for these experiments was 50 mm s^-1^.

Gain versus frequency data from the laser vibrometer were analysed in Origin software (v. 8; see Supporting Methods). The frequency at the centre of the fundamental mode resonant peak (*f* in Hz) was used to calculate the wavespeed (*C* in m s^–1^; Equation (3), using the silk length (*L* in m).


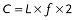
(3)

Ballistic high-rate methods are described elsewhere.[16] Spider silk wavespeed data are measured at a low pre-stress on the fiber (∼10 MPa) at an impact velocity of 220 m s^-1^. For nylon and silkworm silk, the wavespeeds were taken from Drodge et al.[16]

For the transverse and longitudinal wavespeed theoretical calculations, *E* is taken as the quasi-static modulus for CuBe wire (120 GPa; independent of pre-stress), whilst for nylon, silkworm silk, major and minor ampullate silk, *E* is taken from previously published storage modulus data (where minor ampullate silk has an unknown relationship to pre-stress).[18,33] Cross-sectional areas are measured for each material in a Scanning Electron Microscope (SEM; Neoscope 2000, Nikon Instruments UK; Supporting Methods).

## Supporting Information

As a service to our authors and readers, this journal provides supporting information supplied by the authors. Such materials are peer reviewed and may be re-organized for online delivery, but are not copy-edited or typeset. Technical support issues arising from supporting information (other than missing files) should be addressed to the authors.
