# Supplementary material for: The Speed of Sound in Silk: Linking Material Performance to Biological Function
Source: Adv Mater. 2014 Jun 6;26(30):5179–83. doi: 10.1002/adma.201401027 (PMC4140601; doi:10.1002/adma.201401027)
Supplement: Supplementary file 1 — Supplementary [file adma0026-5179-SD1.pdf]

Copyright WILEY-VCH Verlag GmbH & Co. KGaA, 69469 Weinheim, Germany, 2014.

ADVANCED MATERIALS

Supporting Information

for *Adv. Mater.,* DOI: 10.1002/adma.201401027

The Speed of Sound in Silk: Linking Material Performance to Biological Function

Beth Mortimer, Shira D. Gordon, Chris Holland, Clive R. Siviour, Fritz Vollrath, and James F. C. Windmill*

Copyright WILEY-VCH Verlag GmbH & Co. KGaA, 69469 Weinheim, Germany, 2013.

Supporting Information

The Speed of Sound in Silk: Linking Material Performance to Biological Function

Beth Mortimer, Shira D. Gordon, Chris Holland, Clive R. Siviour, Fritz Vollrath and James F. C. Windmill*

Supporting Discussion

Given the experimental confirmation of the physical basis of the sonic properties (Figure 1), signalling consistency of spider silks compared to other types of materials can be further explored by analysing the shape of the resonant peaks as determined by laser vibrometry (Supporting Figure S3). The peak width and height give information on the bandwidth of resonance and on the influence of internal and external damping, where more damping would lead to wider and flatter peaks.[1] The resonant peak area reflects the capacity for vibration information transfer.

Data given in Supporting Figure S4 show that, surprisingly, tension has no significant effect on peak shape. This may be explained by the small displacements used here. Higher displacements are expected to lead to internal damping due to plastic flow of polymer chains, causing a flattening of peak height.[2] Internal damping will be exacerbated by high tensions, not reached here due to limitations of the tensile stage (Supporting Figure S5). Internal damping is expected to affect longitudinal more than transverse waves, because it directly affects modulus, on which the wavespeed depends (Equation (1)).

The resonant peak area for longitudinal waves is approximately consistent for all the materials tested (Supporting Figure S3e). Affecting both wave types, the polymers show wider peaks than the metal alloy as there is an increased distribution of structures within polymers, more consistent in the nylon than in the silks.[2] In contrast, the resonant peak area

for transverse waves is not consistent between materials, where spider silks have a larger peak area (Supporting Figure S3f). The inconsistency stems from the additional factor of external damping acting from air resistance on transverse waves. This is supported by the sensitivity of transverse resonant peak width to fiber diameter and to the number of fibers present. Thinner spider silks show broader peaks, explained by external air damping effects, where smaller fiber diameter leads to a larger relative damping factor, broadening the resonant peak.[3] Importantly, the thinner spider silks additionally have higher amplitude peaks as they couple more readily with the air relative to their mass per unit area.

Importantly, multiple fibers of spider silk show the broadest transverse wave bandwidth out of all materials tested. Increased peak width is due to differential sharing of the load between different fibers, leading to diverging transverse wavespeeds. Divergence is enhanced by including fibers of different diameters and types, such as filaments of both major and minor ampullate silks. Peak broadening could be a property useful for the spiders, as resonance can occur over a broader frequency range. The animal could increase transverse resonant peak area by combining multiple fiber types in a web or even a single thread (as can be found in some web radial threads),[4] thus increasing capacity for information transfer.

Another important factor affecting signalling consistency of materials is the sensitivity of wavespeed to frequency, known as dispersion. Theory suggests internal and external factors will cause a small amount of dispersion by damping the harmonic motion for either wave type.[1] To investigate the relationship between wavespeed and frequency experimentally, transverse resonance of silkworm silk specimens of different lengths were measured (Supporting Figure S2). Transverse wavespeed is approximately constant with changing frequency, but wavespeed is slowed down by c. 100 m s^{-1} at low frequencies (<1 kHz, note log scale), although lower frequencies could not be tested due to practical

difficulties of working with long fibers. Interestingly, this fits in with trends seen in plants, which are dispersive at low frequencies, but non-dispersive at higher frequencies.[5]

Theory suggests dispersion will have a greater effect on longitudinal than transverse waves, as the modulus term that directly affects longitudinal wavespeed (Equation (1)) is sensitive to frequency in polymers (log relationship).[6] This frequency dependence may slightly decrease wavespeed for longitudinal waves at low frequencies, which could explain in part previous observations of slower wave propagation speeds at low forced frequencies.[7] However, it should be noted that the overall effect of dispersion for any wave type will be very small, so wavespeeds are only marginally sensitive to frequency.

Overall, the resonant peak shapes (Supporting Figure S3 and S4) and the dispersion data (Supporting Figure S2) highlight the importance of damping on the consistency of signal transmission.

Supporting Methods

Sample preparation

Spiders of different sizes (larger are older) were reeled, where silk diameter scales with spider size: 'Big', 'Medium' and 'Small' diameters of single major ampullate silks are 6.97, 4.23 and 3.38 μm respectively.

All transverse tests used 12.5 mm frames. For the 12.5 mm spider silk specimens, silk was reeled onto a spool, then glued using cyanoacrylate onto dividers before fixing onto frames.[8] For the 180 mm spider silk specimens, the spider was reeled at the desired speed directly onto the frames. Silkworm silks were additionally mounted onto 5 mm, 50 mm and 180 mm frames for transverse tests. Specimens for longitudinal tests were 180 mm for the

silks, 140 mm for nylon and 220 mm for CuBe wire; different lengths were used to keep the resonance at similar frequencies.

Sample characterization

In order to identify an appropriate range of tensions for the fibers during vibrational experiments, the materials were tested under quasi-static tension (Supporting Figure S5). All fibers were tested at a rate of 1.5 mm min^{-1} until failure occurred (5 N load cell, model 5512, Instron, UK).

For the transverse experiments, the load cell was engaged and constant tension was applied during the experiments. For the longitudinal experiments, the load cell was disengaged because it was found to affect fiber vibration, so extension was used to control tension. Above yield, three minutes were left before testing, so the amount of creep was consistent. Between 3 and 7 specimens were tested for each material type, and two measurements were taken per fiber (low then high tension).

Following the transverse vibrometry experiments, the specimens were tested in tension at a deformation rate of 1.5 mm min^{-1} to the full extension of the tensile stage (10 mm; ranges from 5.6 % strain for 180 mm specimens to breaking for 5 mm specimens). These force-extension profiles were aligned with non-paused force-extension curves to give the stress, strain and length of the fibers during the vibrometry experiments. For longitudinal experiments, the extension was used to infer the strain, stress and length using a reference non-paused force-extension curve.

For transverse waves, the strain parameter acts as a correction for the mass per unit length as the fiber is stretched. The storage modulus of minor ampullate silk was taken from Blackledge *et al.* (2006)[9] using the initial storage modulus of 10.5 GPa. The relationship of

minor ampullate's storage modulus with pre-stress is unknown, so the theoretical line Figure 1 is based only on this storage modulus value, and minor ampullate is not present in Figure 2. Densities were taken as follows: silks = 1,325 kg m^{-3} ,[10] nylon = 1,140 kg m^{-3}, CuBe wire = 8,250 kg m^{-3} [densities taken from manufacturer's data sheet for the material].

All samples were imaged for cross-sectional area in a scanning electron microscope (SEM; Neoscope 2000, Nikon Instruments UK), sputter coated for 150 seconds, 18 mA, giving a 12.5 nm coating of gold/palladium (Quorum Technologies SC7620). Fiber diameters were measured to calculate a circular cross-sectional area with the exception of silkworm silk, which has an irregular cross-section. The method to measure silkworm silk area in the SEM is given elsewhere.[11] A solid cross section is assumed in line with common practice as porosity in silk fibers is negligible, evidenced from pictures of silk fiber cross-sections.[11-12]

Laser vibrometry

A diagram of the set-up is given in Supporting Figure S6. For 180 mm silk specimens, a lower range loudspeaker (L20AT, Nikkai) was used from 100-2,000 Hz. For all tests, the displacement amplitude of the silk was measured at several points (15 averages per point) along the length of the fiber to confirm the wave shape and mode of the vibration. Then, just the middle of the fibers was sampled, where the amplitude was greatest.

To smooth the curves, the gain versus frequency data were filtered using Origin's in-built FFT filter in low pass mode, using an ideal filter. The cut-off time in seconds was selected as 0.004. CuBe wire data were not smoothed due to sharper peaks and a greater signal to noise ratio. The column data were then run through Origin's in-built peak-finding algorithm. The outputs were manually checked to ensure that no peaks were missed and to locate the fundamental mode. Any adjoining peaks (peaks with no gaps between them) were treated as a single peak.

The resonant peak shapes were analysed to give peak width, height and area, where transverse wave peak heights and areas were transformed to allow for comparison (see Supporting Figure S3 and S4). The angle of c. 30 ° used for longitudinal measurements is corrected for when working out displacement along the fiber axis. Additionally, the transverse amplitude data were transformed to account for the different diameters of the fibers. For a given pressure, the force per unit length on the fiber drives the vibration, so to account for this, amplitude data are divided by the fiber's diameter. Since the fibers were all the same length, only the force per unit length needs to be considered.

The force on the fiber can be calculated from Equation (4):

$$F = \frac{1}{2} C_D \rho l d v^2 \tag{4}$$

where C_D is the drag coefficient, ρ the density of air, l the length and d the diameter of the silk fiber and v is the speed of the incoming air. As the speed and density of the incoming air and fiber length are equal between materials, and the drag coefficient is comparable between materials due to the fiber shape, the transverse peak height (in nm Pa^{-1}) is divided by only the fiber diameter (in nm) to give a force per unit length (F) for comparison between fibers (in Pa^{-1}). Peak areas are also divided by the fiber diameter to give a similar parameter that can be compared between fibers.

Average total diameter for two fibers of equal size (diameter D) was taken to be D·(3/2); a combination of one big fiber (diameter D) and two smaller fibers (diameter d) was taken as (D+d)·(2/3); and a combination of two big fibers (diameter D) and two smaller fibers (diameter d) was taken as (D+d). These calculations give a diameter that is roughly average and representative between multiple fibers that twist relative to each other, as an accurate calculation of the diameter facing the on-coming sound front from the speakers is not possible.

Supporting Figures

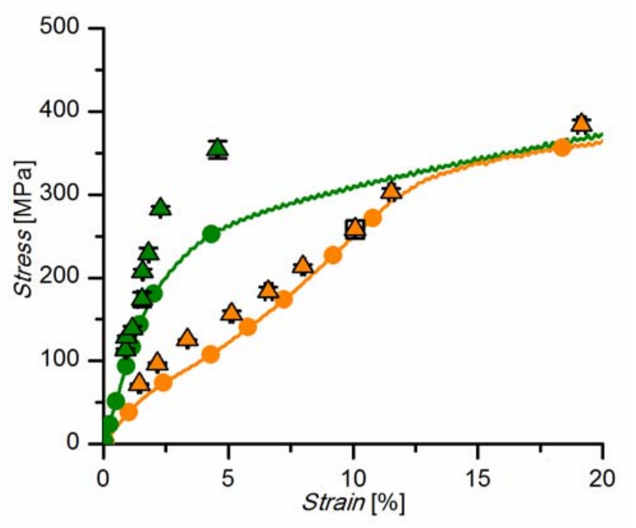

Supporting Figure S1. Quasi-static curve (line) with static pre-stresses (circles) for nylon (orange) and silkworm silk (green). Square scatter points give the ballistic impact high-rate stress-strain co-ordinate following ballistic impact, where an elastic jump from the static pre-stress on the quasi-static curve (circle) is assumed. The error bars give the standard error of the mean for stress and strain for specimens tested at high-rate under identical ballistic impact conditions. Reprinted and minimally modified from Journal of the Mechanics and Physics of Solids, 60/10, Drodge, D. R., Mortimer, B., Holland, C., Siviour, C. R, Ballistic Impact to Access the High-Rate Behaviour of Individual Silk Fibres,, 1710-21., Copyright (2012), with permission from Elsevier.[13]

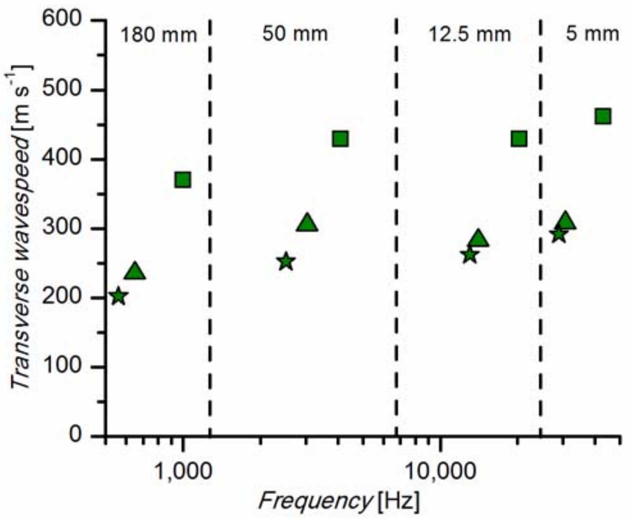

Supporting Figure S2. Dispersion in transverse waves. Frequency (log scale) versus transverse wavespeed for different length silkworm silk specimens at different tensions: squares 170 (± 5.3) MPa, triangles 66 (± 2.4) MPa and stars 49 (± 3.8) MPa. The dashed lines separate the specimens of different lengths, given by the label.

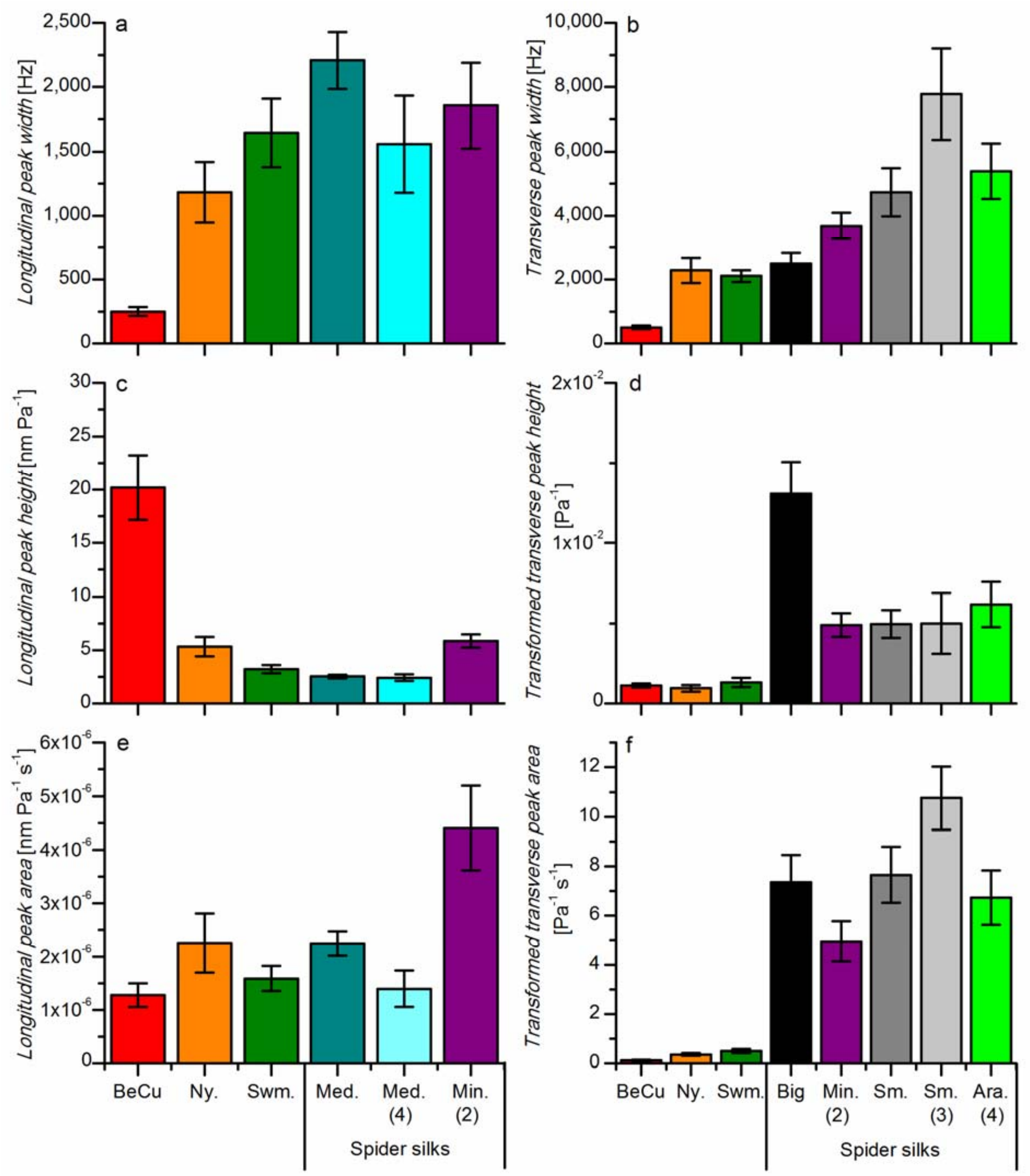

Supporting Figure S3. Resonant peak width data for **a)** longitudinal and **b)** transverse waves; height data for **c)** longitudinal and **d)** transverse waves; area data for **e)** longitudinal and **f)** transverse waves. Height and area outputs are transformed for transverse waves (see Supporting Methods). Given there were no observable effects of tension, average values of all data points for each material type are given where error bars give standard error of the mean. Abbreviations: BeCu is copper beryllium wire, Ny. is nylon; Swm. is silkworm silk where two fibers are bound together; Big, Med. and Sm. refer to the size of the *Nephila* spider being reeled, and so the relative diameters of the silk fibers; Min. is minor ampullate silk and Ara. is *Araneus* silk. Numbers in brackets on the x-axis give the number of fibers present (no number means there is only a single fiber, numbers greater than two include a mix of major and minor ampullate silks).

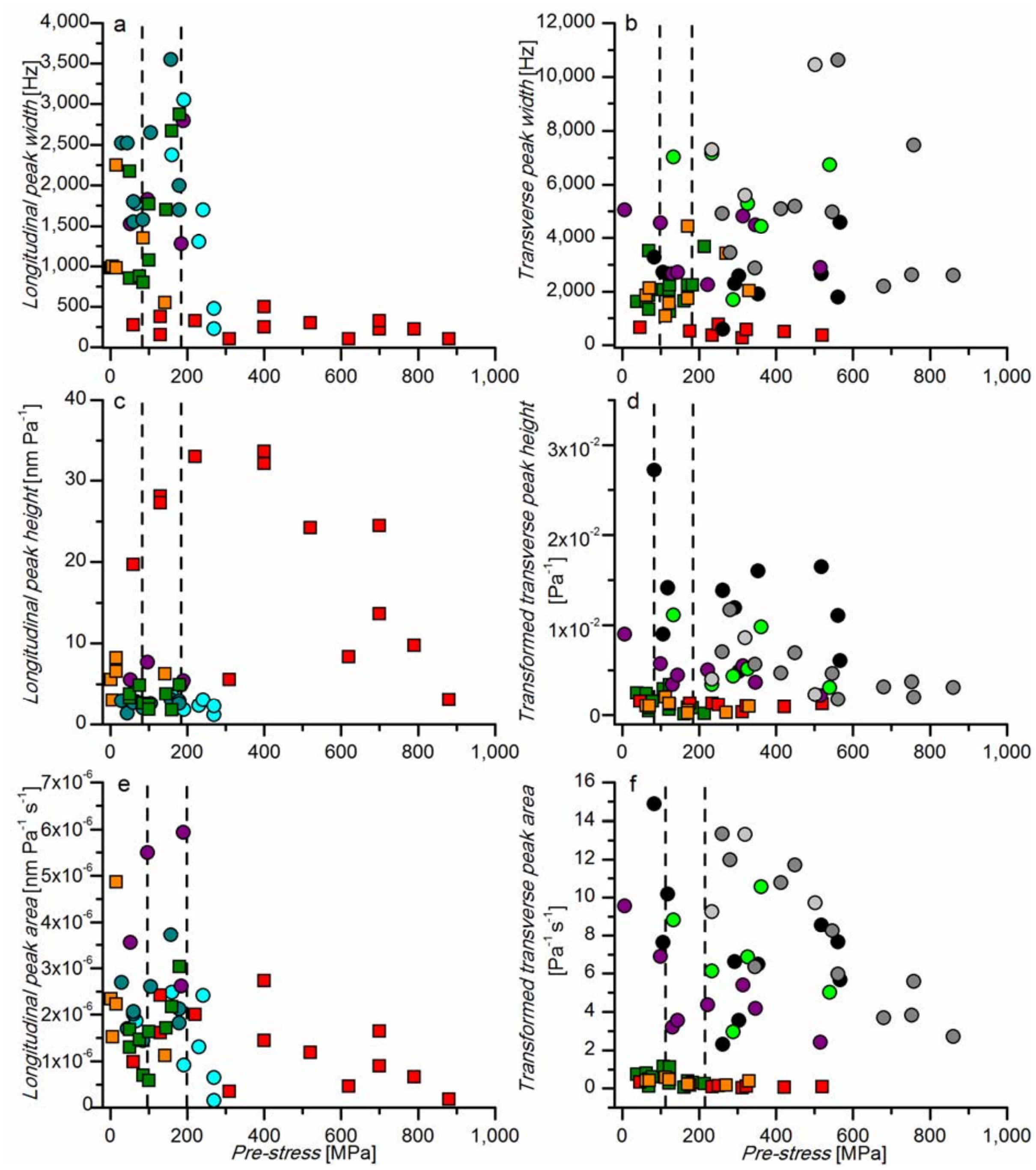

Supporting Figure S4. Resonant peak shapes versus pre-stress. Width data for a) longitudinal waves and b) transverse waves, height data for c) longitudinal waves, and d) transverse waves, and area data for e) longitudinal waves and f) transverse waves. Height and area outputs are transformed for transverse waves (see Supporting Methods). Materials: metal wire (red), nylon (orange) and silkworm silk (dark green) are given by squares; spider silks are given by circles: *Nephila* major ampullate (MA) spider silk (big size, black; medium size, dark cyan; small size, dark grey), *Nephila* minor ampullate (MiA) spider silk (purple), mixture of *Nephila* silks (medium size 2 MA, 2 MiA, cyan; small size 1 MA, 2 MiA, light grey), and *Araneus* spider bundle (2 MA, 2 MiA, green). Dashed lines give the approximate end of the elastic region ~100 MPa and the approximate end of yield ~200 MPa for the polymers.

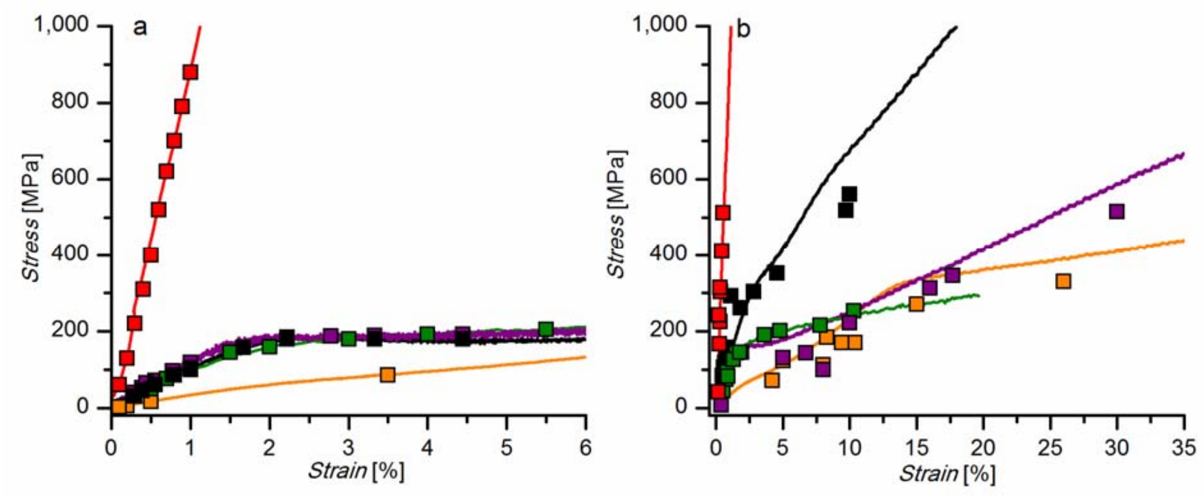

Supporting Figure S5. Vibrometry data stress-strain scatter points with a reference quasi-static stress-strain curve for a) longitudinal specimens (over 140 mm) and b) transverse 12.5 mm specimens. Materials: metal wire (red), silkworm silk (dark green), nylon (orange), *Nephila* major ampullate spider silk (black) and *Nephila* minor ampullate spider silk (purple).

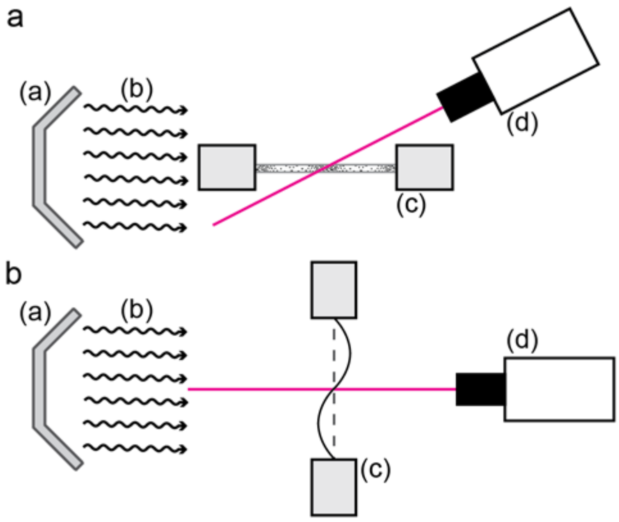

Supporting Figure S6. Experimental set-up for laser vibrometry (view from above) for a) longitudinal and b) transverse tests. A speaker (a) emits sound (b), vibrating the silk clamped between a tensile stage (c) with displacement measured by a laser vibrometer (d).The microphone was placed as close as possible at the intercept between the silk thread and the laser beam, perpendicular to the speaker.

References

[1] I. G. Main, *Vibrations and Waves in Physics*, Cambridge University Press, Cambridge **1993.**

[2] J. D. Ferry, *Viscoelastic Properties of Polymers*, Wiley VCH, New York **1980**.

[3] T. E. Faber, *Fluid Dynamics for Physicists*, Cambridge University Press, Cambridge **1995**.

[4] M. Denny, *J. Exp. Biol.* **1976**, *65*, 483.

[5] J. Casas, C. Magal, J. Sueur, *Proc. R. Soc. B-Biol. Sci.* **2007**, *274*, 1087.

[6] J. Guan, *D. Phil Zoology*, University of Oxford, June, **2013**.

[7] M. A. Landolfa, F. G. Barth, *J. Comp. Physiol. A* **1996**, *179*, 493.

[8] B. Madsen, F. Vollrath, *Naturwissenschaften* **2000**, *87*, 148.

[9] T. A. Blackledge, C. Y. Hayashi, *J. Exp. Biol.* **2006**, *209*, 2452.

[10] J. C. Zemlin, *A Study of the Mechanical Behavior of Spider Silks*, U.S. Army Natick Report AD-684 333, Natick **1968**.

[11] B. Mortimer, C. Holland, F. Vollrath, *Biomacromolecules* **2013**, *14*, 3653.

[12] S. Osaki, *Phys. Rev. Lett.* **2012**, *108*, 154301.

[13] D. R. Drodge, B. Mortimer, C. Holland, C. R. Siviour, *J. Mech. Phys. Solids* **2012**, *60*, 1710.
